# Louse flies (Diptera: Hippoboscidae) of Romania: New records and novel host–parasite and hyperparasites associations

**DOI:** 10.1016/j.ijppaw.2025.101100

**Published:** 2025-06-06

**Authors:** Laura Mlynárová, Peter Manko, Alexandru-Mihai Pintilioaie, Laura-Elena Topală, Martin Hromada, Jozef Oboňa

**Affiliations:** a Department of Ecology, Faculty of Humanities and Natural Sciences, University of Prešov, 17. novembra 1, 081 16, Prešov, Slovakia; b Laboratory of Interdisciplinary Research on the Marine Environment and Marine Terrestrial Atmosphere, “Alexandru Ioan Cuza” University of Iaşi, “Prof. Dr. Ioan Borcea” Marine Biological Station, Nicolae Titulescu str., no. 163, Agigea, Constanţa, Romania; c Doctoral School of Biology, Faculty of Biology, “Alexandru Ioan Cuza” University of Iaşi, Carol I Blvd., No. 20A, 700505, Iaşi, Romania; d Marine Biological Station “Prof. Dr. Ioan Borcea”, Agigea, “Alexandru Ioan Cuza” University of Iaşi, B-dul Carol I, No. 20A, 700 506, Iaşi, Romania; e University of Bucharest, Faculty of Biology, Splaiul Independentei 91-95, Bucharest, 050095, Romania; f Department of Nature Conservation, Faculty of Biological Sciences, University of Zielona Góra, 65417, Zielona Góra, Poland

**Keywords:** Hippoboscids, Faunistics, New record, Phoresy

## Abstract

This study presents records of ectoparasitic flies from the family Hippoboscidae collected in Romania between 2022 and 2024. A total of seven species were recorded, with *Pseudolynchia canariensis* (Macquart in Webb and Berthelot, 1839) representing a new record for the fauna of Romania. To the best of the authors' knowledge, many of the parasite-host associations are new and are previously unpublished. In addition, a graphical network illustrating these associations is provided. This network highlights the frequency and diversity of host-parasite interactions across the study sites. The recorded phoresis of *Guimaraesiella* (Mallophaga) on *Ornithomya avicularia* (Linnaeus, 1758) and the hyperparasites *Hemimyialges macdonaldi* (Evans et al., 1963) and *Myialges anchora* Sergent and Trouessart, 1907 (both Acariformes: Epidermoptidae) on *Ornithoica turdi* (Olivier in Latreille, 1811) are newly documented in Romania.

## Highlights

• The first records of hyperparasitic mites are documented from Romania.
• Host associations were analyzed using the Gephi network.
• New host associations are reported for *Icosta minor*, *Ornithoica turdi*, and *Ornithophila metallica*.
• Phoretic Mallophaga louse was observed attached to *Ornithomya avicularia*.
• The status of non-native species is re-evaluated, and a new record from Romania is presented.

## Graphical abstract

**Figure:**
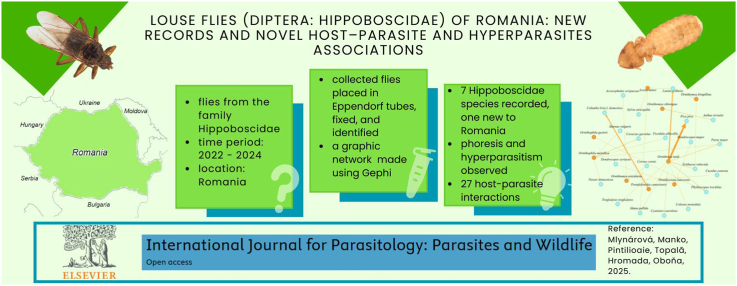

## 1 Introduction

Dipterans from the family Hippoboscidae are obligate ectoparasites of birds and mammals (Rahola et al., 2011). This group has become an increasingly popular subject of research in faunistic, epidemiological, and ecological studies (e.g., Reeves and Lloyd, 2019; Lee et al., 2022; Santolíková et al., 2022; Čisovská Bazsalovicsová et al., 2023; Peña-Espinoza et al., 2023; Tiawsirisup et al., 2023; Wawman, 2024, 2025; Boieiro et al., 2024; Cigler et al., 2024; Frixione et al., 2025; Janiszewska et al., 2025). Thirteen genera comprising more than 213 species have been described worldwide within this family, of which 33 species have been found in Europe, including both bird- and mammal-associated taxa (Dick, 2018; Nartshuk et al., 2018, 2022; Oboňa et al., 2019, 2022; Le Guillou and Chapelin-Viscardi, 2022; Matyukhin et al., 2023; Yatsuk et al., 2023; Keve et al., 2024; González et al., 2024).

In Romania, occurrence of 15 hippoboscid species has been confirmed by Oboňa et al. (2023, 2025), who contributed new faunistic records and compiled previously published data (Thalhammer, 1896; Fleck, 1904; Pârvu and Chimişliu, 1982; Ursu and Pavel, 1993; Pârvu, 2003, 2005; Lazăr et al., 2017; Mihalca et al., 2019; Salvetti et al., 2020). Additional references relevant to the Romanian hippoboscid fauna were identified through a comprehensive literature review, this review also helped clarify previously ambiguous or unpublished records (e.g., Walker and Rotherham, 2010; Rehbein and Mihalca, 2020). Furthermore, personal communication with Steffen Rehbein facilitated the inclusion of an earlier record reported by Vasilescu (1970), who reported the presence of *Lipoptena cervi* (Linnaeus, 1758) - a species not listed in the synthesis by Oboňa et al. (2023). This, previously overlooked record, along with the first report of *Pseudolynchia canariensis* (Macquart in Webb and Berthelot, 1839), increases the known species richness of Hippoboscidae in Romania from 15 to 17, highlighting the importance of re-evaluating historical data in light recent findings.

The study aims to document the diversity and distribution of ectoparasitic dipterans of the family Hippoboscidae recorded in Romania between 2022 and 2024. It also investigates previously unreported host–parasite associations and presents graphical representations of these interactions. Particular attention is given to cases of phoresy, in which smaller arthropods use hippoboscid flies as transport hosts, and hyperparasitism, involving organisms that parasitize the hippoboscids themselves. These findings contribute to a more comprehensive understanding of parasite ecology and transmission dynamics in the region.

## 2 Material and methods

### 2.1 Sample collection

The material of 57 dipterans was collected in five different localities in the "Dunele Marine de la Agigea" Natural Reserve, Biruința, Gârliciu, Leghin and Poiana Teiului in Romania during the seasons 2022–2024 (See Table 1).

**Table 1:** Summary of localities and bird hosts of louse flies analyzed in the current work.

Locality	No. of birds	Latitude	Longitude	Altitude (m a.s.l.)
Dunele Marine de la Agigea" Natural Reserve	28	44°05′11.2"N	28°38′28.2"E	10
Biruinta	3	43°59′37.9"N	28°31′28.8"E	38
Gârliciu (near)	1	44°47′29.7"N	28°05′57.7"E	14
Leghin	1	47°14′44.7"N	26°12′53.9"E	496
Poiana Teiului	1	47°06′53.8"N	25°55′30.8"E	519

Legend: No. of birds – number of infected individuals; m a.s.l. – altitude above mean sea level.

The ‘Dunele Marine de la Agigea’ (sea dunes of Agigea) represent the only natural sea sand dunes left on the entire Romanian coastline outside the Danube Delta in the southeastern Romania. The Biruința and Gârliciu are villages situated in the Constanța County in southeaster Romania. The Leghin village and the Poiana Teiului commune are localized in the Neamț County in northeastern Romania. Details on geographical coordinates are summarized in Table 1.

The fly specimens were collected directly from the birds (the host is mentioned in the paper), or they were found inside the bird ringing room or on humans (the host of the fly cannot be identified).

The collected hippoboscids were placed in Eppendorf tubes, fixed in 96 % ethanol, and then identified using the identification key to Oboňa et al. (2022). The material is deposited at the Department of Ecology, Faculty of Humanities and Natural Sciences, University of Prešov in Slovakia. The main focus on the hosts follows Maa (1969b). The Mallophaga were identified according to Gustafsson et al. (2018) and the Acariformes: Epidermoptidae were identified according to Hill et al. (1967), taking into account Mironov et al. (2005).

References to GBIF online resources are provided for each species.

### 2.2 Data analysis

In order to create the species interaction network (for more details see Mlynárová et al., 2024 for details), qualitative data on the hippoboscid flies collected in the field and data from Oboňa et al. (2023) and their associated bird hosts were analyzed using the Gephi network exploration and manipulation software (https://gephi.org/). In addition to basic information on presence and absence on the host, quantitative data were also used. The developed modules can import, visualize, spatialize, filter, manipulate, and export all types of networks. The visualization module uses a special 3D rendering engine to dispel graphs in real time (Bastian et al., 2009). The typical dataset in Gephi consists of nodes (species in our study) and lines (associations) that connecting these nodes and is represented visually as a graph. To convey the strength or weight of the relationship between nodes, edges have been customized by adjusting their thickness and colour. Thicker or darker edges indicate stronger associations, while thinner or lighter edges indicate weaker associations.

## 3 Results

### 3.1 Taxonomic survey of fauna

Family: Hippoboscidae.

Tribe: Lipoptenini Speiser, 1908.

*Lipoptena fortisetosa* Maa, 1969.

**Published records**: Pârvu (2005), Lazăr et al. (2017), Salvetti et al. (2020).

**Material examined**: Moldova region, Neamț County, Leghin, August 8, 2023, 2 ♀♀, host: unknown.

GBIF database: https://www.gbif.org/species/1638565.

Tribe: Ornithomyini Costa, 1846.

*Icosta minor* (Bigot in Thomson, 1858).

**Published record**: Oboňa et al. (2023).

**Material examined**: "Dunele Marine de la Agigea" Natural Reserve, April 28, 2023, 1 ♀, host: *Sylvia atricapilla* (Linnaeus, 1758) male; May 14, 2024, 1 ♀, host: *Lanius collurio* Linnaeus, 1758.

GBIF database: https://www.gbif.org/species/4516470.

*Ornithoica turdi* (Olivier in Latreille, 1811).

**Published record**: Oboňa et al. (2023).

**Fig. 1:**
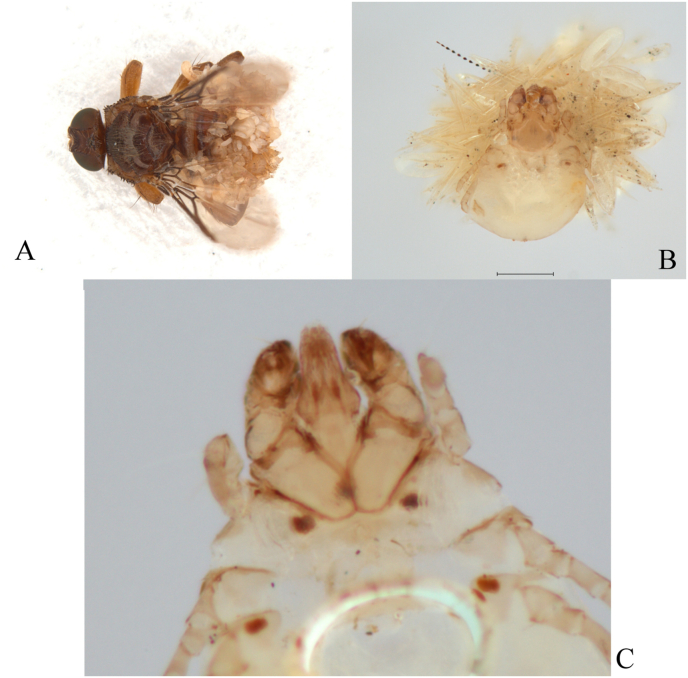
(A) Female of *Ornithoica turdi* (Olivier in Latreille, 1811) with hyperparasite Acariformes: Epidermoptidae, collected from *Parus major* Linnaeus, 1758. (B) Female of *Hemimyialges macdonaldi* (Evans et al., 1963) with eggs, and (C) detail of *H. macdonaldi* (scale: 0.1 mm).

**Fig. 2:**
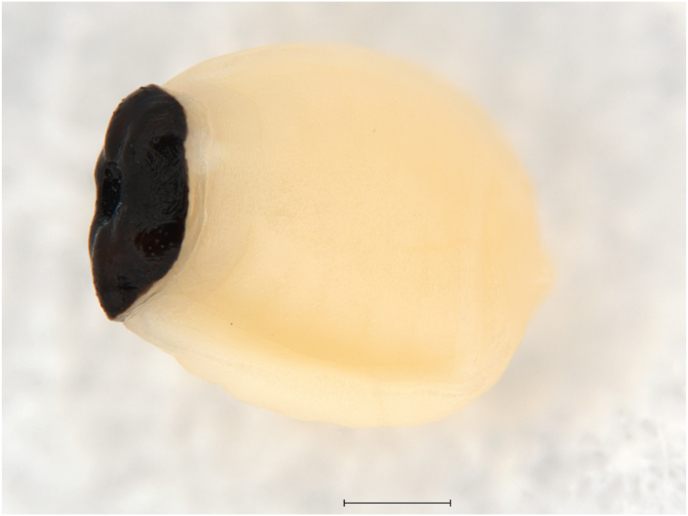
Newly born larva of *Ornithoica turdi* (Olivier in Latreille, 1811) collected from *Lanius collurio* Linnaeus, 1758 (scale: 0.1 mm).

**Material examined**: "Dunele Marine de la Agigea" Natural Reserve, November 24, 2022, 1 ♀, host: *Parus major* Linnaeus, 1758 (fly with hyperparasite Acariformes: Epidermoptidae: *Hemimyialges macdonaldi* (Evans et al., 1963) on abdomen (Fig. 1A–C); August 12, 2023, 1 ♀, host: *L. collurio*; August 21, 2023, 1 ♀, host: *Coloeus monedula* (Linnaeus, 1758); August 29, 2023, 1 ♀, host: *L. collurio* (with the newly born larva – Fig. 2); September 12, 2023, 1 ♀, host: *Phylloscopus trochilus* (Linnaeus, 1758); October 12, 2023, 1 ♀, host: *Cyanistes caeruleus* (Linnaeus, 1758) (ring number: x157584); October 28, 2023, 1 ♀, host: *Troglodytes troglodytes* (Linnaeus, 1758); October 30, 2023, 1 ♀, host: *Erithacus rubecula* (Linnaeus, 1758) (ring number: R300353); November 20, 2023, 1 ♀, host: *E. rubecula* (ring number: R302469); June 20, 2024, 4 ♂♂, 12 ♀♀, host: *Pica pica* (Linnaeus, 1758); June 20, 2024, 1 ♀, host: *P. major*; August 2, 2024, 1 ♀, host: *L. collurio*; August 3, 2024, 1 ♀, host: unknow; August 11, 2024, 4 ♀♀ (fly with hyperparasite Acariformes: Epidermoptidae: *Myialges anchora* Sergent and Trouessart, 1907 on abdomen) host: *L. collurio*.

GBIF database: https://www.gbif.org/species/11015538.

Superorder: ∗Acariformes.

Family: Epidermoptidae.

*Hemimyialges macdonaldi* (Evans et al., 1963).

GBIF database: https://www.gbif.org/species/12306595.

*Myialges anchora* Sergent and Trouessart, 1907.

GBIF database: https://www.gbif.org/species/4547612.

*Ornithophila metallica* (Schiner, 1864).

**Published record**: Oboňa et al. (2023).

**Material examined**: "Dunele Marine de la Agigea" Natural Reserve, May 1, 2023, 1 ♀ host: *Ficedula albicollis* (Temminck, 1815); June 10, 2024, 1 ♀, *Sturnus vulgaris* Linnaeus, 1758 juv.; Biruinta, July 12, 2023, 1 ♂, *Coracias garrulus* Linnaeus, 1758 (fledgling); July 16, 2024, 1 ♀, *C. garrulus.* Gârliciu, August 5, 2024, 1 ♂, host: *Passer domesticus* (Linnaeus, 1758).

GBIF database: https://www.gbif.org/species/1638964.

*Ornithomya avicularia* (Linnaeus, 1758).

**Published records**: Thalhammer (1896), Pârvu (2003), Oboňa et al. (2023).

**Fig. 3:**
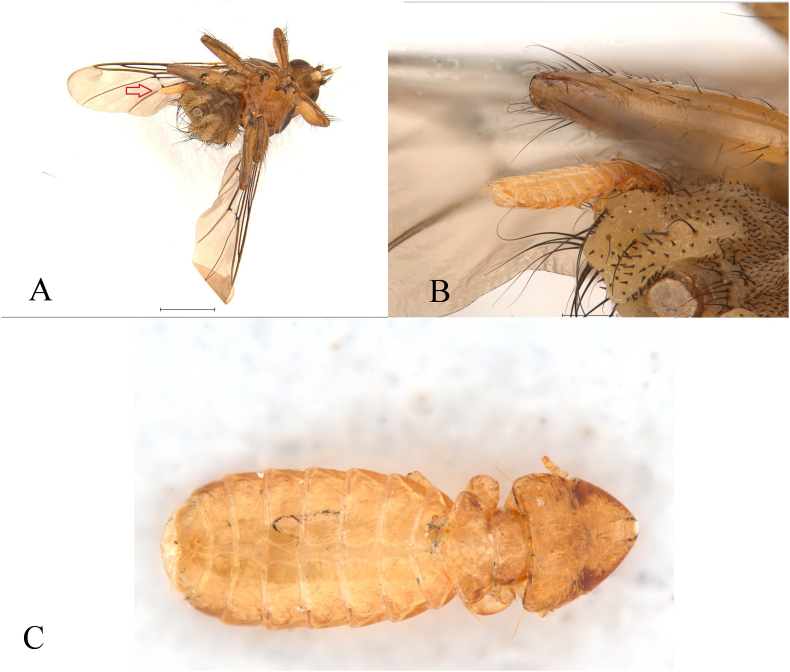
(A) Female of *Ornithomya avicularia* (Linnaeus, 1758) from an unknown host, (B) with attached phoretic Mallophaga. (C) Detail of Mallophaga: genus *Guimaraesiella* (scales: A – 1 mm, B – 0.1 mm).

**Material examined**: Poiana Teiului, August 7, 2023, 1 ♀, host: unknown (fly with phoretic Mallophaga: genus *Guimaraesiella,* Fig. 3A–C); "Dunele Marine de la Agigea" Natural Reserve, August 22, 2023, 1 ♀, host: *Acrocephalus scirpaceus* (Hermann, 1804); June 10, 2023, 1 ♂, host: *Cuculus canorus* Linnaeus, 1758.

GBIF database: https://www.gbif.org/species/1638360.

*Ornithomya chloropus* (Rondani, 1878).

**Published record**: Oboňa et al. (2023).

**Material examined**: "Dunele Marine de la Agigea" Natural Reserve, September 6, 2023, 1 ♀, host: *Anthus trivialis* (Linnaeus, 1758) (ring number: R300658).

GBIF database: https://www.gbif.org/species/1638374.

*Pseudolynchia canariensis* (Macquart in Webb and Berthelot, 1839).

**Fig. 4:**
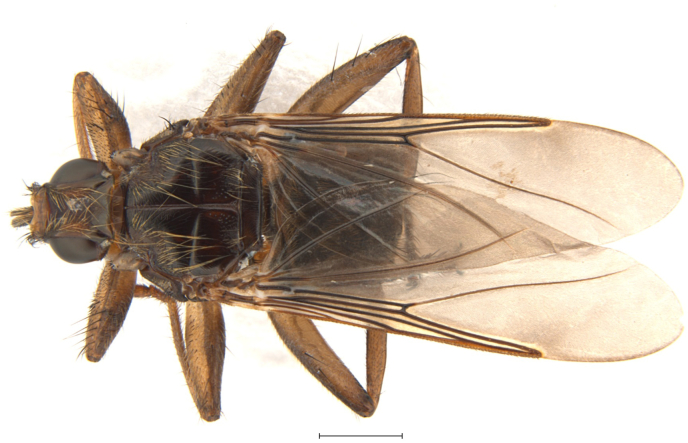
*Pseudolynchia canariensis* (Macquart in Webb and Berthelot, 1839) male, collected from an unknown host in the "Dunele Marine de la Agigea" Natural Reserve, southeaster Romania. Dorsal view (scale: 1 mm).

**Material examined**: "Dunele Marine de la Agigea" Natural Reserve, September 12, 2023, 1 ♂, host: unknown; October 16, 2023, 1 ♂ (Fig. 4), host: unknown; November 1, 2023, 2 ♂♂, 1 ♀, host: *Columba livia* f. *domestica* Gmelin, 1789; September 12, 2024, 2 ♂♂, 2 ♀♀, 10.2024, 1 ♀, host: unknown; November 15, 2024, 1 ♂, host: unknown.

GBIF database: https://www.gbif.org/species/1639231.

### 3.2 Host parasites association

**Fig. 5:**
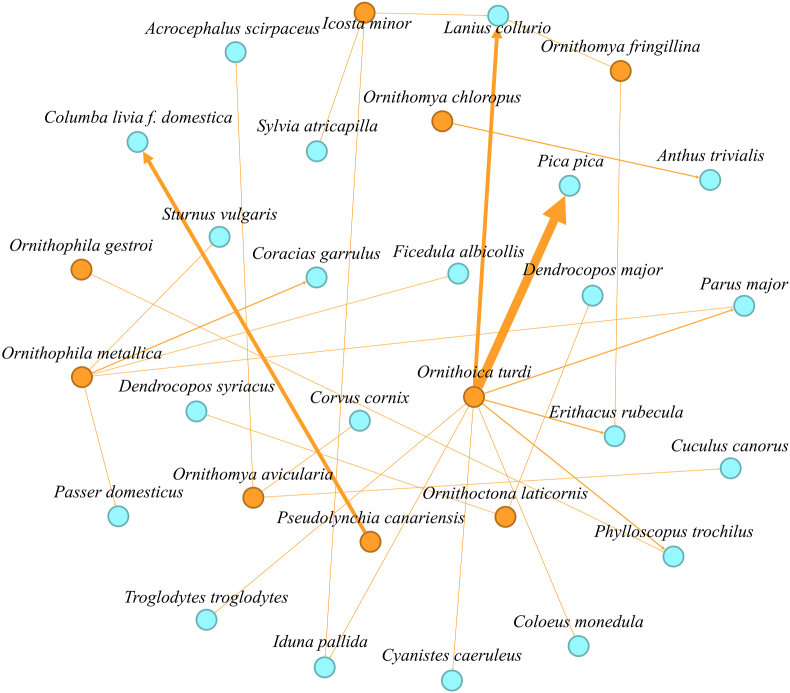
Species interaction network of hippoboscid flies and their associated bird hosts. Orange dots represent hippoboscid parasites, and blue dots represent avian hosts. The thickness of the lines indicates the relative frequency of the interactions.

According to Oboňa et al. (2022), the status of some non-native species in Romania is still uncertain, particularly for *O. turdi* and *O. gestroi*. Based on the species interaction network analysis (see Fig. 5), the status of *O. turdi* could be re-evaluated as native for this region.

In total, 17 different bird species and several unknown avian hosts were examined in this study. Seven louse fly species were detected; one (*L. fortisetosa*) from the tribe Lipoptenini, and six (*I. minor*, *O. turdi*, *O. metallica*, *O. avicularia*, *O. chloropus* and *P. canariensis*) from the tribe Ornithomyini. Two hyperparasitic species *H. macdonaldi* and *M. anchora,* were detected in *P. major* and *L. collurio*, respectively.

In the species interaction network analysis, 27 host-parasite associations have been documented between bird (host) and parasite (Hippoboscidae) species from five different localities (Fig. 5). The highest relative frequency of interactions was found between *O. turdi* and *P. pica*, as well as *O. turdi* and *L. collurio*, and *P. canariensis* and *C. livia* f. *domestica* (Fig. 5, thicker orange arrows). The highest number of associations with different host species (n = 9) was detected in *O. turdi*, followed by *O. metallica*, which was associated with 5 bird hosts. *Icosta minor* and *O. avicularia* each had 3 associations. Two associations were recorded for *O. fringillina* and *O. laticornis,* while only one association was recorded for *O. chloropus*, *O. gestroi,* and *P. canariensis*.

## 4 Discussion

### 4.1 Taxonomic summary and ecological discussion of the recorded fauna

A taxonomic survey of the family Hippoboscidae on birds in Romania conducted between 2022 and 2024 revealed representatives from two tribes: Lipoptenini and Ornithomyini. This survey builds upon earlier records and reveals both native and non-native species.

Within Lipoptenini*,* the invasive species *L*. *fortisetosa* was recorded. This non-native louse fly, originally of East Asian origin, is currently widespread in several parts of Europe (Andreani et al., 2019, 2021; Kurina et al., 2019; Oboňa et al., 2022).

In total, six species from the tribe Ornithomyini were identified: *Icosta minor*, a small-bodied species, is recorded in the Afrotropical region and the Mediterranean basin (Trilar & Krčmar, 2005; Sychra et al., 2020; Jentzsch et al., 2021). In Romania, we recorded new host associations with *S. atricapilla* and *L. collurio*, both migratory, thereby broadening its documented host range (cf. Jentzsch et al., 2021). *Ornithomya turdi* is widely distributed extending across the Afrotropical and southern Palearctic regions, with a recent surge in European records (Droz and Haenni, 2011; Zittra et al., 2020; Gaponov and Tewelde, 2020; Kock, 2000; Keve et al., 2024). In Romania, it was found parasitizing five new avian hosts: *C. caeruleus, C. monedula, P. major, P. pica,* and *P. trochilus*. Notably, 16 individuals were recovered from a single *P. pica*, the highest infestation level known for this fly. This unusually high load may indicate localized abundance or host susceptibility. Previously, *Circus macrourus* (S. G. Gmelin, 1770) in Uganda held the record with 10 individuals (Maa, 1966). The polyxenous, non-native species *O. metallica* is known across the southern Palearctic, Afrotropical, Oriental, and Australasian regions (Krišovský et al., 2024; Nartshuk and Matyukhin, 2019; Lehikoinen et al., 2021; Lee et al., 2022). Previously associated with *Sturnus* and *Coracias* spp. (Maa, 1969b), our study documents previously unreported hosts, *F. albicollis* and *P. domesticus*. *Ornithomya avicularia* and *O. chloropus* are both common Palearctic louse flies, primarily parasitizing passerines but occasionally found on other birds (Krišovský et al., 2024; Keve et al., 2024). These species were frequently encountered, indicating their broad host range and ecological adaptability. Their presence across multiple habitats reinforces their status as core members of Central European hipposcid assemblages. *Pseudolynchia canariensis*, a cosmopolitan species primarily associated with domestic pigeons, represents a new national record in Romania. This species is known from the Ethiopian, Mediterranean, Oriental, Palearctic, Nearctic, and Neotropical regions (Maa, 1969a, 1969b).

In association with louse flies, mites of the family Epidermoptidae (Acariformes) were also found. Both mite species were found on *O. turdi*, which was collected from a host individuals of *L*. *collurio* and *P. major*. These findings provide evidence of complex multitrophic interactions. *Hemimyialges macdonaldi*, originally described as *Myialges macdonaldi* Evans et al., 1963, is a highly specialized ectoparasite of hippoboscid flies (Evans et al., 1963; Mironov et al., 2005). It exhibits a dual parasitic strategy: fertilized females attach to the fly for oviposition, while other life stages parasitize birds – particularly *C. caeruleus* (Philips, 2000; Krantz and Walter, 2009). Larvae hatch on the fly and migrate to the avian host (Evans et al., 1963). Such host strategy exemplifies the intricacy of avian ectoparasite systems. The second species, *Myialges anchora*, is a cosmopolitan hyperparasite, previously documented in Slovakia on *O. turdi* associated with *L. collurio* (Hromada et al., 2022). This study provides the first verified documentation of both mite species in Romanian avifauna.

Additionally, lice of the genus *Guimaraesiella* (Mallophaga) were found phoretically attached to *O*. *avicularia*. This cosmopolitan and morphologically variable genus includes species with broad host ranges, many of which are phoretic on hippoboscid flies (Bartlow et al., 2016; Gustafsson and Bush, 2017; Bush et al., 2016). This supports growing evidence of louse dispersal via hippoboscid vectors. Given currents limitations in species-level identification, further morphological or molecular work is needed to confirm identities. Integrative taxonomic approaches will be essential for resolving these ambiguities.

### 4.2 Ecological and taxonomic insights into host–parasite associations

This study expands the current understanding of hippoboscid and hyperparasitic mite diversity in Romania by documenting seven louse fly species and two hyperparasitic mite taxa across a limited geographic and temporal scope suggesting unexpectedly high ectoparasite richness relative to prior national records. This may reflect previously underreported diversity rather than recent faunal shifts.

A particularly noteworthy finding of this study is the documentation of *O*. *turdi* in association with nine different avian host species in Romania, underscoring its broad ecological adaptability. This expanded host range and distribution call into question the previous classification of *O. turdi* as a non-native species in the region (Oboňa et al., 2022). While the possibility of recent introduction cannot be fully excluded, the increasing number of records across Europe (Zittra et al., 2020; Gaponov and Tewelde, 2020) suggests that *O. turdi* may be an established native or naturalized species in Romania. These distributional changes are likely influenced by factors such as migratory bird movements and climate-driven range shifts (Dufour et al., 2020), warranting further investigation into its population dynamics and ecological status. Anthropogenic habitat change may also facilitate range expansion.

Equally significant is the observed shift in species dominance within the region's hippoboscid community. While *O*. *avicularia* and *O. fringillina* are known to dominate host-parasite interactions in Central Europe (Mlynárová et al., 2024), our data indicate that *O. turdi* and *O*. *metallica* now appear to dominate the Romanian hippoboscid assemblage. This pattern suggests regional variation in species composition and host associations, possibly driven by ecological, climatic, or biogeographical factors. Understanding these shifts is crucial for elucidating the dynamics of hippoboscid fly populations and their potential impact on local avian hosts.

The exceptionally high infestation intensity observed (16 *O*. *turdi* individuals on a single *P*. *pica* host) raises questions about the factors influencing parasite load. Host-specific traits such as behaviour and body size, as well as local environmental conditions, may play critical roles in facilitating such dense infestations. This finding underscores the need for detailed investigations into host-parasite interactions, particularly in ecologically transitional zones like the Agigea dunes, where environmental heterogeneity may promote complex parasite dynamics.

A key advancement presented by this study is the first confirmed records of two hyperparasitic mite species, *H*. *macdonaldi* and *M*. *anchora*, in Romania. These mites exhibit complex life cycles, alternating between parasitizing hippoboscid flies and their avian hosts (Philips, 2000; Mironov et al., 2005). Their presence may have important epidemiological implications, especially within migratory bird populations, as these mites could function as both vectors and reservoirs for additional pathogens (Bartlow et al., 2016). However, direct pathogen screening was beyond the scope of this study. 10.13039/100014337Furthermore, the detection of phoretic *Guimaraesiella* lice on *O*. *avicularia* supports accumulating evidence that hippoboscid flies serve as phoretic transport vectors for lice (Gustafsson and Bush, 2017), and potentially contribute to the transmission of other associated pathogens.

The species interaction network constructed in this study revealed 27 unique host-parasite associations, including several novel interactions not previously documented in the literature. Notably, associations involving *I*. *minor*, *O*. *turdi*, and *O*. *metallica* underscore the dynamic and largely underexplored nature of avian ectoparasite ecology in Eastern Europe. These associations may have biogeographical or conservation relevance. Given that many of the host species are migratory, it is plausible that some interactions represent transient associations facilitated by long-distance movements across biogeographical regions (Su et al., 2022). These findings highlight the importance of long-term, multi-site monitoring to determine the stability, specificity, and ecological significance of these associations.

From a methodological point of view, the study faced several limitations, which should be taken into account when interpreting host specificity. The opportunistic nature of some collections, such as specimens obtained from birds handled during ringing operations or from human observers, may have introduced minor sampling biases that affect the perceived prevalence or distribution of certain species. Moreover, the presence of unidentified or partially identified avian hosts limited the ability to accurately assess host specificity and ecological fidelity. Despite these constraints, the integration of original field data with existing records, analyzed through species interaction network frameworks, provides a robust framework to characterize parasite community structure and assessing the potential for host switching and pathogen transmission.

Collectively, the results of this study highlight the ecological significance of hippoboscid flies and their associated microfauna within Romanian avifauna, including their roles as ecoparasites, phoretic hosts and substrates for hyperparasites. These ectoparasites, along with their hyperparasites and phoretic organisms, represent an underexplored but ecologically meaningful component of avian communities, with potential implications for both biodiversity and disease ecology, especially in ecotones and migratory corridors. To build on these findings, future research should focus on longitudinal monitoring to capture seasonal and interannual dynamics, quantify impacts on host fitness, and implement pathogen screening protocols to assess the potential for zoonotic transmission. Continued, coordinated efforts by Romanian ornithologists and parasitologists will be instrumental in revealing the complexity of host-parasite interactions and their consequences for avian and broader ecosystem health. Given the increasing movement of wildlife across biogeographic boundaries – whether due to migration, habitat change, or climate shifts – understanding these networks is critical for anticipating and mitigating emerging infectious disease risks (Runghen et al., 2021; Su et al., 2022; Mlynárová et al., 2024).

## CRediT authorship contribution statement

**Laura Mlynárová:** Writing – review & editing, Writing – original draft, Visualization, Validation, Project administration, Data curation, Conceptualization. **Peter Manko:** Writing – review & editing, Writing – original draft, Visualization, Conceptualization. **Alexandru-Mihai Pintilioaie:** Writing – review & editing, Writing – original draft, Visualization, Validation, Data curation, Conceptualization. **Laura-Elena Topală:** Writing – review & editing, Writing – original draft. **Martin Hromada:** Writing – review & editing, Writing – original draft. **Jozef Oboňa:** Writing – review & editing, Writing – original draft, Visualization, Validation, Methodology, Conceptualization.

## Funding

This work was supported by the 10.13039/501100005357Slovak Research and Development Agency under contract No. APVV-22-0440. The work of L. M. was funded by the EU NextGenerationEU through the Recovery and Resilience Plan for Slovakia under the project No. 09I03-03-V05-00006. The research conducted by A.-M. Pintilioaie was supported by the Operational Program Competitiveness 2014–2020, Axis 1, under POC/448/1/1 Research infrastructure projects for public R&D institutions/Sections F 2018, through the Research Center with Integrated Techniques for Atmospheric Aerosol Investigation in Romania (RECENT AIR) project, under grant agreement MySMIS no. 127324.

## Declaration of competing interests

The authors declare that they have no conflict of interest regarding the publication of this manuscript.
